# The Structure and Function of Research Ethics Committees in Africa: A Case Study

**DOI:** 10.1371/journal.pmed.0040003

**Published:** 2007-01-23

**Authors:** Nancy E Kass, Adnan Ali Hyder, Ademola Ajuwon, John Appiah-Poku, Nicola Barsdorf, Dya Eldin Elsayed, Mantoa Mokhachane, Bavon Mupenda, Paul Ndebele, Godwin Ndossi, Bornwell Sikateyo, Godfrey Tangwa, Paulina Tindana

## Abstract

Kass and colleagues explore the strengths and weaknesses of research ethics committees in Africa.

According to international guidelines [1,2] and several nations' laws [3–5], research with humans requires independent ethics committee review. In the United States, committees are called institutional review boards (IRBs) [6]; elsewhere they generally are called research ethics committees (RECs). Committees are designed to: provide third party review, thereby minimizing conflicts of interest; protect the welfare of research participants through attention to risks, benefits, and informed consent; and avoid exploitation of vulnerable individuals and populations.

Most literature examining RECs comes from wealthier countries. One US study found “serious concerns” with the quality of 14% of IRB reviews [7]. Another found that IRBs focused predominantly on consent documentation, spending less time examining voluntariness, selection of participants, and risk [8]. Many US [9–15] and international [16–18] studies have found that different research ethics committees reach different conclusions when reviewing the same study.

Several scholars and advisory bodies have made recommendations to address challenges facing US IRBs [19–22]. However, there has been little research examining procedures, strengths, and challenges of RECs in developing countries. Two case reports describe disagreements between host and sponsoring country RECs [23,24], and an international survey reports differences in sponsoring and host country reviews [25]. Three articles describe RECs within one country (Turkey [26], Granada [27], and Sudan [28]), and five within a larger region.

Rivera described 20 RECs in Latin America, finding that only 45% had standard operating procedures and that members had limited training [29]. Coker examined RECs in Central and Eastern Europe [30]. Ten countries had national committees, most committees included non-medical members, and three provided training. The World Health Organization's (WHO) Southeast Asian Regional Office, finding that only some of the 16 respondents had national RECs, called for capacity development in the area of research ethics [31].

Most literature examining research ethics committees comes from wealthier countries.

The WHO African Regional Office found that 36% of member countries had no REC. In the countries that did have RECs, most RECs met monthly, five met quarterly, and one never met [32]. Finally, Milford examined African RECs' resource needs in the context of HIV vaccine trial preparedness, finding that 97% believed African RECs had inadequate training in ethics and HIV vaccine trials and 80% believed African RECs had inadequate training in health research ethics.

Additional information on how African RECs function, including their staffing, operating procedures, strengths, and challenges would be useful for African and international researchers working within Africa, and for growing efforts to enhance ethics capacity on this vast continent. We therefore used a case study approach to shed light on the structure and functioning of RECs in Africa.

## Methods

The Johns Hopkins Bloomberg School of Public Health received a training grant from the Fogarty International Center in 2000 to train three African professionals in bioethics each year [33]. Several of these professionals explicitly seek to increase the scholarly and administrative capacity of their African RECs. In 2004, program faculty and trainees created a structured questionnaire to document the history, composition, functioning, financing, strengths, and challenges of RECs with which the trainees were affiliated. Questionnaires were completed by e-mail. Follow up e-mails clarified responses. Data were entered into Microsoft Excel and tabulated. Trainees and faculty met for two days in 2005 to refine concepts and work on the manuscript.

## Results of Our Case Study

Eleven of the 12 trainees who attended the program in 2001–2004 collaborated. Nine had personal experience on one or more African REC. Another trainee secured information from her institution's REC; one contributed no data. One trainee worked with two committees in his country; another worked with two committees from two countries. Twice, two trainees from the same country were affiliated with different RECs. Thus, twelve RECs were included in this case study from nine African countries: Democratic Republic of the Congo, Ghana (2), Kenya, Nigeria, South Africa (2), Sudan (2), Tanzania, Zambia, and Zimbabwe.

### History of research ethics committees.

The oldest committee was from South Africa, established in 1967. The REC of the Medical Research Council of Zimbabwe was formed in 1974 but had intermittent functioning until 1992, when it became more formally established. Two RECs began in the 1980s; eight were started within the last five years, including two (Kenya and Democratic Republic of the Congo) created by the trainee the year before data collection.

Six of the 12 RECs had Federal Wide Assurances (FWAs) from the US government, an indication that the institution had received US research funds or collaborated with US institutions [34]. Two RECs were established as a requirement of international collaboration. The remaining RECs were established because of a recognized need for independent ethics review. Trainees' efforts were responsible for existing or pending FWAs of three African institutions.

### Composition.

RECs ranged from nine to 31 members. One included only physicians and scientists, while most had clinicians, social scientists, economists, nutritionists, pharmacists, statisticians, pastors, and lawyers. Ten had lay or non-scientist members; two did not (see Table 1). One required that a third of the members should be lay persons, including a traditional chief and representatives from local organizations. Another asked the local community to nominate a community member. None required gender balance, but all consciously included both men and women.

**Table 1:**
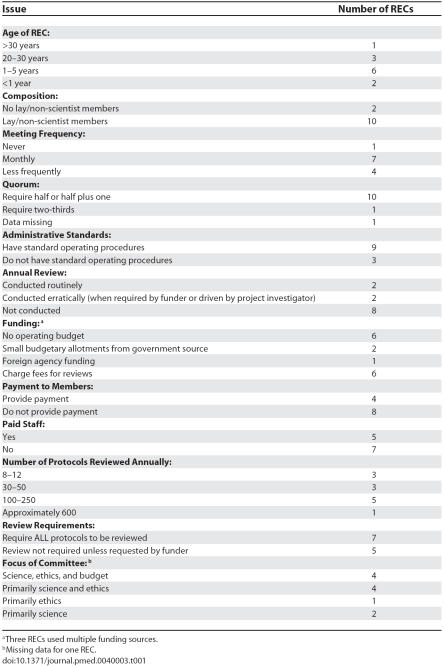
Composition of RECs in Case Study

### REC meetings.

One REC recently stopped meeting in person; reviews were conducted by the chair or individual members. All other committees met in person: two met irregularly, based on need; another met twice per year or as needed; one met every two months; and seven met monthly.

All committees (except the one that did not meet) had requirements for quorum (half, or half plus one). One required two-thirds attendance. Meeting quorum, in general, was not difficult. One trainee said members were committed to duties; two said meetings were scheduled in advance or on weekends. Two said quorum was a problem. One described significant member turnover; another said busy members had problems with punctuality and attendance.

### Training of REC members.

Two committees had members with no training. Six RECs had received training only since the Johns Hopkins University (JHU)–Fogarty trainee returned and provided it. Four RECs had individual members who attended external workshops; one committee conducted Good Clinical Practice courses semi-annually.

### Conflicts of interest.

All RECs required that members be excused if their protocol was under review. Other potential conflicts were raised, however, which may be harder to manage. Two discussed conflicts posed when a departmental colleague had a protocol under review. One said such reviews were sent to another department, even to a department with less expertise, to avoid conflicts. Another described unease voicing objections when fellow members' protocols were reviewed, fearing being labeled unfriendly. Another believed community members were loathe to reject protocols because studies bring employment. Another said protocols bring income to the institution and sometimes questions were not raised so projects could clear quickly.

### Procedural and administrative issues.

Most RECs had basic administrative capabilities, although the REC that no longer met in person lacked any administrative infrastructure. Two RECs lacked standard operating procedures. Nine had such procedures in place, five of which had been written by the trainee upon returning to Africa. All eleven RECs that met kept minutes.

All RECs had a mechanism for reviewing research project amendments to approved studies, although most did not require a review for study changes or amendments. In four RECs, the JHU–Fogarty trainee created the amendment mechanism. Of the 12 RECs, two routinely conducted annual reviews (both instituted this practice after the trainee returned to Africa); two conducted annual reviews when required by an external funder or driven by the principal investigator; and eight did not conduct annual reviews.

### Finances.

All trainees said REC funding was a challenge. Three had no operating funds whatsoever. For the other nine, funding came solely or in combination from government (2), foreign agencies (1), and/or fees for reviews (6). Fees for review varied greatly. One REC used a “sliding scale,” charging US$5 for proposals submitted by students, US$10 for studies submitted by post-graduate trainees, and US$20 for all other research proposals. Another did not charge for institutional applications, but required US$365 for external applications and US$585 for industry studies. Some used a “fixed fee” structure, such as US$100 for all applications or 1% of the study's budget, once funded. All RECs benefited from “in-kind” donations of institutional resources, such as space, photocopying, mail distribution, and services of staff with other responsibilities.

Eight RECs did not pay members (though some reimbursed travel); four paid a “sitting allowance.” Five RECs had paid staff; seven did not. RECs that paid staff all had budgetary allotments or charged fees.

### REC review.

The number of protocols reviewed per year varied tremendously. Three RECs reviewed eight to 12 protocols per year, three reviewed 30–50, five reviewed 100–250, and one reviewed 600 per year. Two RECs with small portfolios only reviewed internally funded protocols. Most reviewed a mixture of internal and external projects.

Seven RECs required all protocols to be reviewed, although two started this policy only after the JHU–Fogarty trainee returned to the institution. The five other RECs only reviewed research when required by the funder. Review time generally corresponded to the frequency with which the REC met. Most completed reviews in one to two months, ranging from two weeks to more than three months. Four RECs looked equally at science, ethics, and budget, while another four reviewed science and ethics, but not budget. Two spent little time on ethics, while another focused almost exclusively on ethics as another committee reviewed the science.

### Strengths.

Trainees mentioned several strengths of their RECs. First, the creation of so many new committees is a strength in itself. Also, many committees have at least a few members who received some training in ethics, through the REC, the JHU–Fogarty trainee, or external workshops. Several trainees mentioned that their REC has a reputation with sponsors for integrity and/or that the REC provides useful feedback to researchers.

### Challenges.

Inadequate training and funding consistently were mentioned as the biggest challenges. These scholars acknowledged significant time and effort for member training. Reviewers were often poorly equipped to review according to ethics criteria, which led to a disproportionate focus on the science. Trainees mentioned inadequate training of staff and administrators in REC procedures; one trainee raised the issue that RECs have weak monitoring systems due to funding constraints.

Budget constraints were mentioned by nearly everyone. Running an REC is expensive, and one trainee suggested that for this reason poor countries will simply avoid the creation of a REC unless required. Another said that governments must be made aware of the importance of research ethics to convince them to fund RECs. Several mentioned that REC members had multiple responsibilities and thus, they would be more committed if they could be paid, especially since serving on the REC might actually deny them income they would otherwise have received for that time. One REC had no stationery, space, computers, or communication facilities. In another, a foreign investigator donated $200 for stationery supplies when the REC started, but there were no other funds for staff or infrastructure. An REC started by the JHU–Fogarty trainee used the trainee's personal laptop for its official business. Trainees also used their reentry grants provided by the JHU program to help enhance REC infrastructure.

Another challenge was the tendency of a few RECs to “rubber stamp” approvals in order to secure international funding. Related to this challenge, a couple of trainees raised a concern about REC independence. One said outsiders, researchers, and politicians could interfere in the REC process, and another said the “culture of corruption” is prevalent in some parts of Africa, which could affect the integrity of the committee. In some regions, investigators could engage in “IRB shopping,” whereby they could submit their protocol to a new REC if it was rejected by a first. A few trainees were concerned about possible abuse of the expedited review option in their RECs, as expedited reviews do not incur the delay and expense of convening a full committee meeting. Two specifically mentioned a lack of national guidelines and local operating procedures as a challenge to good work. Another voiced a concern that institutions would often select “top management” individuals to be members who might not have appropriate skills or time.

### Suggestions.

Given the challenges raised, it was not surprising to hear trainees suggest the need for more training, funding, independence, and political commitment to improve REC functioning. In addition, innovative suggestions also emerged: training workshops on how to interpret ethics principles in light of local norms; public outreach programs about research; creation of networks of African RECs to share materials, resources, and capacity building; creation of mechanisms to facilitate communication between host and sponsor country RECs; joint meetings between REC members and investigators to brainstorm solutions to shared challenges; human rights advocacy to help enhance participants' and researchers' awareness about rights in research; and more empirical research on ethics and African research.

## Discussion

This case study reports on the experience of ten African professionals with 12 African RECs. These 12 RECs represent a range of experiences, from a committee formed 30 years ago to two recent ones. All, to greater or lesser extents, are functional, although one never meets as a committee. All cite the need for additional training, more attention to *ethics* issues, and more funding for staffing, transportation, and supplies.

Many challenges described here are not unique to African RECs. Wealthier countries, too, have heard criticism about inadequate funding, staffing, and training of committees [35–40]. Poor countries, however, inevitably feel these needs more acutely. Further, additional challenges may arise from resources being limited. We heard of institutions or community members exerting pressure to approve research that would bring jobs, infrastructure development, money, and intellectual cache to the local setting. Kilama suggested that poverty itself is a threat to independence, since poverty can blind researchers, participants, and RECs alike to any problems in studies that bring jobs, medicines, or prestige to a community [41]. Challenges to people's integrity may be more typical where individuals can expedite or bypass usual procedures through informal transfer of funds, as occurs in some countries.

External mandates often were the impetus for a committee forming and, in some cases, contributed start-up resources. While some committees still only review externally sponsored projects, others used external requirements as a catalyst to create a conscientious committee, committed to ethics review, training, and integrity. Absent the external mandate, changes may have happened more slowly.

### Encouraging lessons.

Positive lessons can be drawn from this case study. First, research ethics review is increasingly routine in Africa. More African institutions require and are equipped to provide review, all but one of the committees in this case study meet in person, and membership is relatively diverse. There are growing opportunities in Africa for training in Good Clinical Practice and research methodology. Increasingly, African investigators submit to international journals that require REC review as a condition of publication; African journals now, also, generally require REC review of published studies [42,43], and a special meeting of the Forum for African Medical Editors in 2005 developed further guidelines for journal submission and review, including guidelines related to ethics [42]. Nonetheless, several of these committees are new, and some were created by the trainee. In the future, other researchers may start an African collaboration, find no RECs exist locally, and will need to facilitate creation of one. More guidance exists to assist in this task, but it can appear somewhat daunting [44].

Second, these experiences suggest committees become more stable, equipped, and trained over time. Thus, some challenges described may reflect how new most African RECs are. Committees with the longest history are the most established with regard to procedures, funding, and staffing. One trainee described his REC focusing almost entirely on science when first created, with community members deferring to scientific ones. Over time, members gained training and experience, and reviews began to include more ethics.

Third, this case study suggests individuals can make a difference. RECs included here were not random: a professional associated with them had just completed intensive training in research ethics. Nonetheless, with limited funds and variable institutional support, a small number of individuals created two RECs, others created and implemented standard operating procedures, review forms, and regular review where none existed, and most now provide training for members, researchers, and/or the public.

Further progress likely will involve a confluence of funders' requirements for review, institutional commitments, and individual contributions. Indeed, successful change requires systemic commitment. One individual cannot effect long-term change without institutional support, which is more likely with national requirements for review [45]. National policies are more likely to be developed when international funders, aid agencies, and journals establish that RECs are required and review must be the norm. National and institutional commitment must be set as policy and implemented through influx of resources for RECs.

To make committees' work meaningful, however, there must be a commitment, as many have suggested, to training and better resources. We join others calling for a shared library of resources, model standard operating procedures [46], model consent forms, and copies of training presentations; fortunately, such resources increasingly are available through the Internet. African professionals must find means to access continuing ethics education [41,47]. Challenging ethics dilemmas will always arise in research; those tasked with resolving them will need ongoing support and training to navigate reasonable solutions.

### Limitations of our case study.

This case study has several limitations. The data are self-reported, through the lens of individuals who received intensive training in research ethics. Thus, their views may reflect more sophisticated understanding of how RECs should function than other REC members might provide. Further, the capacity of RECs, as reported, was often recently enhanced due to the efforts of the JHU–Fogarty trainee. Most new African RECs presumably are not started with these resources and intellectual capacity development, so the speed with which new RECs develop procedures and skills for ethics review may happen more slowly.

This report describes 12 RECs in Africa. It does not claim to be representative of African RECs as a whole. Further, this case study examined REC functioning but does not attempt to draw a conclusion about how ethical research is in Africa. Even the most conscientious REC review does not guarantee a well-executed study. Without study monitoring, it is impossible to know the relationship between REC quality and the quality of approved research [48,49].

## Conclusion

This case study examines the history, operations, strengths, and challenges of 12 African RECs. We hope this will help researchers working in Africa better understand the landscape of ethics review and help funders target resources for capacity development in a continent where health research is so critical to development, and local responsibility for research functions is critical for research.

## Supporting Information

Alternative Language Abstract S1 French Translation of the Abstract by BM (31 KB DOC).

## Abbreviations

FWA: Federal Wide Assurance
IRB: institutional review board
JHU: Johns Hopkins University
REC: research ethics committee
WHO: World Health Organization
