# Supplementary material for: The Structure and Function of Research Ethics Committees in Africa: A Case Study
Source: PLoS Med. 2007 Jan 23;4(1):e3. doi: 10.1371/journal.pmed.0040003 (PMC1779815; doi:10.1371/journal.pmed.0040003)
Supplement: Supplementary file 1 [file pmed.0040003.sd001.doc]

**French version**

**Translated by Bavon Mupenda**

**Structure et fonction des Comités d’éthique de la recherche en Afrique : Une étude de cas**

Résumé

Beaucoup de guides de procédure et de lois exigent qu’une évaluation éthique indépendante soit faite pour une recherche. Aux États-unis, ces comités sont connus sous le nom de Conseils Institutionnels de Révision, en Anglais (Institutional Review Boards ou IRB en sigle). Ailleurs, ces comités sont souvent appelés Comités d’Éthique de la Recherche (CER). Des comités sont donc établis pour donner une évaluation exempte de parti pris en vue de minimiser les conflits d’intérêt, de protéger le bien-être des participants à la recherche par une attention particulière sur les risques, les avantages et le consentement éclairé, mais aussi pour empêcher l’exploitation des individus et des populations vulnérables. Alors qu’il existe une littérature sur les CER des pays les plus nantis, il y a très peu d’études qui examinent la structure, la fonction, les forces et les défis des comités africains.

Le programme de formation des Africains en Éthique de la recherche, conjointement organisé par Johns Hopkins et Fogarty donne une formation annuelle en bioéthique et en éthique de la recherche aux professionnels africains. En 2004, le corps enseignant et les apprenants avaient créé un questionnaire sur l’histoire, la composition, le fonctionnement, le financement, les forces et les défis des CER auxquels appartiennent les apprenants. Il en est ressorti qu’un comité avait été formé en 1967, tandis que huit n’ont commencé qu’il y a cinq ans. Six de douze CER avaient une convention avec le gouvernement des États-unis, convention appelée Federal Wide Assurance (FWA), ce qui suggère qu’il y a eu une collaboration dans le passé avec les États-unis. Deux ont été créés parce que c’était un préalable pour une collaboration internationale. Le nombre de membres des comités varie entre 9 et 31. Tous les comités, à l’exception d’un seul, se réunissent par personnes physiques et tous se réunissent mensuellement ou tous les deux mois. Apparemment, deux comités n’avaient pas de formation, tandis que six n’avaient reçu de formation que depuis le retour des apprenants de JHU-Fogarty qui se sont chargés de donner cette formation. Le financement était un défi pour tous les CER. Trois d’entre eux n’avaient pas de fonds pour fonctionner et six faisaient payer des frais pour chaque évaluation qu’ils faisaient

Les forces notées comprenaient le fait que maintenant des CER existent en Afrique en plus grand nombre qu’avant et, si l’on peut ainsi dire, l’évaluation des projets de recherche est perçue comme faisant partie de la culture des professionnels locaux dans beaucoup de régions. La plupart de comités ont au moins quelques membres avec une formation en éthique. Les comités ayant une plus longue histoire sont les plus réputés en matière de procédures, de financement et des membres. De nouveaux comités sont en train d’être établis, en développant ainsi une infrastructure digne de confiance, les unes en un temps record.

.

Parmi les défis, on peut citer une formation insuffisante et un manque de financement. Les évaluateurs se sentent parfois mal équipés pour faire l’évaluation selon les critères éthiques. Cela fait que, souvent, ils se concentrent plus disproportionnellement sur les aspects scientifiques des travaux. Les membres des CER sont aussi partagés entre de multiples responsabilités qui se télescopent. Certains comités ont peu ou pas du tout d’infrastructure pour les soutenir dans leur exercice. Quelques apprenants avaient fait mention des possibilités de conflits lorsqu’il s’agissait d’évaluer les protocoles.

Plusieurs suggestions ont été faites dans le sens de l’amélioration des CER africains. Elles vont des recommandations pour davantage de formations, de financement et d’indépendance à des programmes publics de descente sur le terrain au sujet de la recherche et la création des réseaux en vue de s’échanger du matériel entre les CER.
